# Diabetes-specific formula as meal replacement for individuals with type 2 diabetes mellitus and overweight or obesity: a Singapore expert consensus

**DOI:** 10.3389/fnut.2026.1810277

**Published:** 2026-05-13

**Authors:** Chin Meng Khoo, Matthew Tan, Soo Kiang Eng, Choon Kit Leong, Yong Mong Bee, Kwang Wei Tham, Kalpana Bhaskaran, Yik Voon Lee, Gladys Hooi Chuan Wong, Soo Ling Chan, Phong Ching Lee

**Affiliations:** 1 Division of Endocrinology, National University Hospital, Singapore, Singapore; 2 Diabetes and Endocrine Care, Farrer Park Hospital, Singapore, Singapore; 3 Central-North Primary Care Network, Singapore, Singapore; 4 Mission (Hougang) Medical Clinic, Singapore, Singapore; 5 Department of Endocrinology, Singapore General Hospital, Singapore, Singapore; 6 Department of Endocrinology, Woodlands Hospital, Singapore, Singapore; 7 Glycemic Index Research Unit, Temasek Polytechnic, Singapore, Singapore; 8 Singapore Nutrition and Dietetics Association, Singapore, Singapore; 9 Lee & Tan Family Clinic and Surgery, Singapore, Singapore; 10 Nutrition & Dietetics, Khoo Teck Puat Hospital, Singapore, Singapore; 11 Division of Endocrinology, Ng Teng Fong General Hospital, Singapore, Singapore

**Keywords:** blood glucose, consensus, diabetes mellitus, type 2/diet therapy, diabetes-specific formula, meal replacement, nutrition therapy, obesity/diet therapy

## Abstract

**Introduction:**

In Singapore, only one-quarter of patients with type 2 diabetes mellitus and overweight/obesity in the primary care setting are achieving clinical targets with a food-first approach to medical nutrition therapy. Diabetes-specific formula as meal replacement (DSF-MR) provides an evidence-based escalation of care but remains underutilised in practice. We developed expert consensus recommendations to support the use of DSF-MR in Singapore and across Asia.

**Methods:**

A multidisciplinary 11-member advisory board guided a multicomponent study that included an online healthcare professional survey, a narrative literature review, and structured consensus development. Survey insights shaped research questions and the narrative review, and collated findings were subsequently discussed at an advisory board meeting. Practical recommendations emerging from the meeting were finalised through two rounds of blinded online voting (>70% agreement) and then consolidated into a clinical algorithm for practice.

**Results:**

Eighteen practical, evidence-based recommendations achieved consensus. Collectively, they outline the clinical role of DSF-MR across three key applications: improving glycaemic control, supporting diabetes remission, and preserving muscle mass. Recommendations also address patient selection, integration within holistic care approaches, monitoring, and improving adherence, with the aim of optimising outcomes with DSF-MR.

**Conclusion:**

DSF-MR is supported by evidence as a versatile dietary intervention that can be integrated across the diabetes care continuum. These expert consensus recommendations address critical gaps in current practice and provide clear and actionable guidance for the use of DSF-MR in routine diabetes care across primary care and specialist settings in Singapore.

## 1 Introduction

Excess bodyweight is a prevalent comorbidity among individuals with type 2 diabetes mellitus (T2DM) (1–3). In individuals with overweight or obesity, weight loss has a positive impact on glycemic management (4, 5). The American Diabetes Association (ADA) 2026 guidelines recommend sustained weight loss of at least 5–7% from baseline for individuals with prediabetes, T2DM, overweight, or obesity to improve glycaemia and other cardiovascular risk factors such as hypertension and dyslipidaemia (4). Moreover, a sustained loss of 10% of body weight can potentially induce remission of T2DM (5).

With glucagon-like peptide-1 (GLP-1) receptor agonists (RAs) receiving regulatory approval to treat both T2DM and obesity (6), non-pharmaceutical interventions such as medical nutritional therapy (MNT) – which rely on sustained and patient-led behavioural modification to achieve metabolic benefits – may be overlooked or underutilised in clinical practice. However, MNT remains a cornerstone of treatment guidelines across the continuum of dysglycaemia, from prediabetes to longstanding T2DM. MNT is also essential for the sustained effectiveness of concomitant pharmacological approaches to both T2DM and overweight/obesity (4, 7). MNT is also critical for addressing growing concerns over the reported adverse effects of GLP-1 RAs, including nausea, vomiting, diarrhoea, lean muscle loss and micronutrient deficiencies (4, 8–13).

We conducted a healthcare professional (HCP) survey (*n* = 48) and found that Singaporean patients with T2DM and overweight/obesity [defined as body mass index (BMI) of 23.0–32.4 kg/m^2^] commonly receive nutritional advice to reduce their carbohydrate intake and avoid sugary beverages. Despite this, less than a quarter of patients seen by general practitioners (GPs) were meeting glycaemic control and weight loss targets. For many patients, there was a need for escalation of MNT strategy to improve metabolic parameters and achieve clinical goals.

Diabetes-specific formula (DSF) is a specialised, complete, and balanced nutritional product designed to support the dietary management of T2DM. DSFs facilitate calorie and carbohydrate intake (typically 1.0–1.6 kcal/mL) through a low-glycaemic carbohydrate blend. Additionally, DSFs provide a lipid profile optimised for cardiometabolic health, essential vitamins and minerals, and both soluble and insoluble dietary fibre to improve glycaemic control, support gut health, and enhance satiety (14–18). DSFs are typically formulated with high biological value protein (e.g., whey, soy and casein), which exhibit high digestibility and amino acid availability, thereby supporting muscle protein synthesis and preservation (19–22). DSFs may also comprise ingredients that enhance insulin sensitivity and macronutrient metabolism (e.g., inositol, chromium) (14–18). Under the guidance of an HCP, DSF may be incorporated into the diet in several ways. In this manuscript, meal replacement (MR) refers to the substitution of main meals with a DSF (DSF-MR) as part of a structured hypocaloric diet (typically 1,200–1800 kcal/day), to support energy restriction while retaining protein and micronutrient intake (4, 23–26). When DSF-MR comprises an entire meal, it is said to be full MR (23, 24). Partial MR is when DSF is the primary component of a meal and complemented by healthy food options (e.g., non-starchy vegetables, low glycaemic index fruits, lean protein, whole grains) (26). Lastly, total diet replacement (TDR) refers to the replacement of all meals (≥3 per day) with DSF as part of a very low-calorie diet (~800–1,000 kcal/day), which should be initiated only at the discretion of, and under the supervision of, HCPs (4, 25, 27, 28).

DSF use is endorsed by experts and clinically proven to support glycaemic control, promote quality weight loss, and improve cardiometabolic risk factors in individuals with T2DM and overweight/obesity (14, 26, 29–31). Greater improvements in haemoglobin A1c (HbA1c) and weight loss are observed with more frequent MR and routine use for at least 6 months (23, 24, 26, 29, 32). However, despite its potential, the HCP survey revealed that only 1 in 3 Singaporean GPs recommend DSF-MR to their patients. Another third of GP respondents indicated they lacked sufficient training for providing detailed nutritional advice. Moreover, the majority reported that they had insufficient time to provide nutritional advice.

This manuscript, comprising expert recommendations and a practical treatment algorithm, has been developed to support DSF-MR integration in time-limited consultations, with the aim to bridge existing gaps in clinician training and confidence.

## 2 Materials and methods

This was a multicomponent study comprising a national survey of HCPs, a narrative literature review graded against ADA criteria, and a modified Delphi consensus. The study was helmed by an advisory board of 11 members who specialise in endocrinology, general practice, or dietetics in hospital settings and private practice in Singapore.

To profile the use of MNT and DSF-MR within clinical settings and to identify HCP knowledge and confidence gaps relating to dietary interventions, the authors developed a 24-item online survey (Supplementary Information 1) that was published on the FormsSG digital survey platform in March 2025. To ensure specificity of survey responses, HCPs were instructed to consider only their patients with T2DM and overweight or mild obesity, defined as per World Health Organization criteria for Asian populations (BMI 23.0–27.4 kg/m^2^ and BMI 27.5–32.4 kg/m^2^, respectively). (33) Patients with higher grades of obesity (BMI ≥ 32.5 kg/m^2^), for whom surgical intervention would be indicated, were excluded from survey responses. The authors received 48 anonymous responses to the survey from dietitians, diabetes nurse educators, GPs, and endocrinologists practicing in Singapore (Supplementary Information 2). Results were analysed in April 2025.

Research questions were developed to address the clinical gaps identified through analysis of survey results; these questions (“Who, What, When, Why, and How”) guided the narrative literature review and synthesis of evidence. The scope of the literature review encompassed publications that demonstrated potential benefit of DSF in individuals with both T2DM and overweight/obesity. Studies in which participants received DSF-MR as full MR were prioritised over partial MR, to facilitate faster onset of weight loss and thus more immediate metabolic benefits. Items that were considered relevant for data extraction included study type, inclusion and exclusion criteria, intervention protocol (e.g., number of meals replaced, duration of intervention, daily calorie limit, and comparator/s), baseline characteristics, assessment schedule, ancillary support (such as counselling and exercise), and outcomes (including reductions in HbA1c, fasting blood glucose, body weight, BMI, blood pressure, and medication use). Subsequently, literature summaries were generated for DSF-MR across four topics: glycaemic control and weight management, muscle protection, diabetes remission, and optimising outcomes. Literature was provisionally graded according to the framework offered by the ADA (4), and grades were confirmed through online voting.

An advisory board meeting in May 2025 was used to review the survey results and available evidence, and to provide perspectives on the applicability of this literature to real-world practice in Singapore. Additionally, advisors were asked to provide specific guidance for improving patient adherence, reducing healthcare burden, and optimising multidisciplinary engagement.

Practical recommendations were extracted from the meeting discussions and refined through two rounds of independent, blinded consensus voting using an online platform (i.e., Formsite). For each recommendation, advisors could *A: Agree*, *B: Agree with some revision*, or *C: Disagree*. Advisors could also provide their suggestions for statement modifications as free text. Consensus was defined as >70% agreement (*A*) among the advisors at the final round of voting. Consensus recommendations were then synthesised into a simplified clinical algorithm to facilitate real-world application.

## 3 Results

A set of 18 recommendation statements, pertaining to four key topics, were drafted and refined through two rounds of voting (Table 1; Supplementary Information 3). The following is a narrative review of the literature intended to accompany and provide rationale for the final statements.

**Table 1:** Finalised expert recommendations for use of DSF-MR in individuals with T2DM and overweight/obesity.

Indication/topic	Expert recommendations	Agreement, % (*n* = 11)
DSF-MR for patients with T2DM and overweight/obesity for better glycaemic control	As part of a standard approach to medical nutrition therapy for diabetes care, diabetes-specific formula (DSF) should be recommended as full meal replacement (FMR) to patients with both type 2 diabetes mellitus (T2DM) and overweight/obesity, particularly those who: Are not meeting glycaemic or weight loss targets with previous dietary interventions; those who are reluctant to increase their medication burden for glycaemic control; or those who have health conditions that limit physical activity, making dietary intervention a more central treatment strategy Are unable to comply with a whole food-based approach or previously recommended meal plans, perhaps owing to limited resources, low confidence, limited nutrition literacy or cooking skills; or those who have a busy lifestyle and value convenience Are willing to initiate and sustain DSF-MR for the duration required to achieve the clinical goals set by the healthcare professional	72.7%
	DSF can be used as a full replacement for 1–2 meals per day for ≥3 months in patients with T2DM and overweight/obesity, and as long as required to achieve clinical goals, and may have a greater effect on HbA1c and bodyweight when used for ≥6 months. DSF should ideally be used as part of a hypocaloric diet and in conjunction with a structured exercise regimen.	90.9%
	The benefits of a structured lifestyle intervention for glycaemic control and weight loss – which includes DSF as meal replacement for 1–2 meals/day within a hypocaloric diet – may be more pronounced if recommended alongside motivational interviewing.	100.0%
	To optimise adherence to meal replacement with DSF, as part of a hypocaloric diet, a stepwise approach is recommended. Start with 1 meal replacement/day and gradually increase the number of meal replacements/day, to achieve and sustain the recommended number of servings/day indicated for weight loss or glycaemic goals. Recommended servings per day should be individualised and adjusted according to body mass index at initiation and patient tolerance/motivation.	90.9%
	At initiation, DSF as a replacement for breakfast is most studied and could be more acceptable and convenient to patients than replacing other meals. Meal/s to be replaced with DSF can also be determined by post-prandial glycaemic profiles, prioritising the meal that results in the largest glucose excursion.	90.9%
	Structured short-term patient-led glucose monitoring at initiation of DSF-MR (e.g., tracking blood glucose levels before and after meals), along with maintaining a food diary, could be considered to help patients visualise improvements in glycaemic control and encourage compliance with DSF as a meal replacement. Disclaimer: The use of DSF-MR as full replacement of 1–2 meals per day does not require monitoring for safety purposes, as changes will be gradual. Intensive and/or ongoing monitoring may be indicated for other aspects of patient care, such as medication adjustment and hypoglycaemia prevention.	72.7%
DSF-MR for patients with T2DM and overweight/obesity for muscle protection in the era of new drugs (e.g., GLP-1 receptor agonists [RAs])	Patients with T2DM and overweight/obesity who are receiving GLP-1 RAs can benefit from nutritionally dense, complete, and balanced hypocaloric meals to address the need for glycaemic control, weight loss, and adequate protein for muscle protection while accounting for limited appetite and/or poor dietary habits that can result in nutrient deficiencies.	90.9%
	At GLP-1 RA treatment initiation, a protein intake goal of 1.2–1.5 g/kg/day of high biological value could be achieved with a meal plan that includes DSF as meal replacement for 1–2 meals a day, in addition to protein-rich foods or protein modular supplements. This meal plan will provide a nutritionally dense, complete, and balanced hypocaloric diet to support muscle health and glycaemic control while reducing overall body weight in patients with T2DM and overweight/obesity.	100.0%
	For patients with T2DM and overweight/obesity who are diagnosed with sarcopenia or have experienced rapid weight loss following GLP-1 RA initiation, a protein intake goal of 1.2–1.5 g/kg/day of high biological value could be achieved with a meal plan that includes DSF as meal replacement for 1–2 meals a day, in addition to protein-rich foods or protein modular supplements. This meal plan could provide a nutritionally dense, complete, and balanced hypocaloric diet to restore muscle health (in combination with strength training), improve glycaemic control, and facilitate continued weight loss if clinically indicated.	90.9%
DSF-MR for patients with overweight/obesity and recently diagnosed T2DM to achieve diabetes remission	A diet of 800–1,000 kcal/day for 12 weeks, which could be achieved through TDR with DSF, can help to attain diabetes remission in patients with overweight/obesity and newly diagnosed T2DM (≤6 years). Diabetes remission is defined as HbA1c < 6.5% achieved through intensive behavioural changes and sustained after ≥12 weeks without glucose lowering medications. Disclaimer: TDR must take place under medical supervision Patients who are receiving insulin are not suitable candidates for TDR BMI thresholds considered suitable for TDR initiation may vary by ethnicity.	72.7%
	A 6- to 12-week structured food reintroduction phase, which includes DSF to replace 1 meal a day within a hypocaloric diet, can support glycaemic and weight control after the TDR phase.	90.9%
	A 6-month weight maintenance phase, which may include DSF to replace 1 meal a day within a hypocaloric diet, could help to support glycaemic and weight control after the structured food introduction phase.	90.9%
Optimising outcomes for patients with T2DM and overweight/obesity using DSF-MR for glycaemic control and weight loss	Within a hypocaloric diet or TDR framework, DSF could be accompanied by a portion of non-starchy high-fibre vegetables to support satiety, enhance tolerability of the diet, and improve overall patient satisfaction.	81.8%
	Adherence to a hypocaloric diet, which includes DSF-MR for glycaemic control and weight loss, can be optimised by routine, coordinated, and sustained involvement of a multidisciplinary team (MDT) that includes a doctor, dietitian, physiotherapist, and diabetes nurse educator. MDT members must provide specialised advice and share responsibility for monitoring patient progress.	81.8%
	For patients following a hypocaloric diet, which includes DSF-MR for glycaemic control and weight loss, remote check-ins, combining in-person visits with telehealth, staged follow-up, and shared digital platforms can be utilised to maintain frequent touchpoints with patients without overburdening staff or healthcare resources.	100.0%
	Pre-emptive tapering of insulin or sulfonylureas is recommended for patients who intend to follow a hypocaloric diet. The tapering schedule should be tailored to the individual patient’s medical condition and guided by clinical judgement. Disclaimer: Factors that may influence the tapering schedule include assessment of hypoglycaemia risk, baseline HbA1c, and meal replacement plan. Adjustments to the treatment plan should be made through shared decision-making and guided by close blood glucose monitoring.	81.8%
	Pre-emptive tapering of antihypertensive medications may be considered for patients who intend to follow a hypocaloric diet. The tapering schedule should be tailored to the individual patient’s clinical condition (e.g., through home blood pressure readings), specific antihypertensive medication prescribed, and response to hypocaloric diet (e.g., degree of weight loss).	90.9%
	Regular review of glucose-lowering medication dosage is advised for all patients with T2DM and overweight/obesity who have initiated a hypocaloric diet.	100.0%

### 3.1 DSF-MR for glycaemic control and weight loss

In alignment with existing consensus recommendations (14), the advisory board recommends that DSF-MR be initiated to support glycaemic control and weight loss, as part of a holistic approach to care that is inclusive of a nutrient-dense hypocaloric diet, behavioural counselling, a structured exercise regimen, and multidisciplinary team (MDT) support, in patients with T2DM and overweight/obesity.

There is firm evidence of the efficacy of liquid MRs for glycaemic control and weight loss. In individuals with T2DM and overweight/obesity, meta-analyses have shown that at least 2 weeks’ use of 1–3 liquid MRs daily can improve HbA1c, fasting glucose, body weight, and BMI (29, 32, 34). These benefits were significantly more pronounced when the liquid MR was DSF (29).

After initiating a hypocaloric diet that includes full replacement of 1–2 meals a day with DSF, HbA1c reductions have been demonstrated as early as 3 weeks, with reductions of 0.5–0.9% observed at 3 months for both overweight and obese individuals (23). Reductions in body weight (~1.7 kg), fasting glucose (~1 mmol/L; ~18 mg/dL), and postprandial glucose (~1.7 mmol/L; ~30 mg/dL) have also been observed at 3 months (23, 26). A meta-analysis has demonstrated that longer-term (≥6 months) use of MR can result in even greater improvements to glycaemic control and body composition than shorter interventions (32). This is exemplified by a study that reported weight loss of ~5 kg, BMI reduction of ~2.0 kg/m^2^, and decrease in body fat percentage of ~1.5% at 6 months of DSF-MR use, with 1–2 servings per day (24).

While most individuals with T2DM and overweight/obesity may benefit from DSF-MR (14), this dietary approach should ideally be personalised based on BMI at initiation, clinical targets, and patient preferences. Consider initiating patients with one serving per day to facilitate familiarity and acceptance, gradually increasing as needed to achieve clinical goals. As expected, greater metabolic benefits are seen with two daily servings, in terms of HbA1c, weight loss, BMI, visceral adipose tissue volume, triglycerides, and total cholesterol to high-density lipoprotein ratio, when compared with one serving of DSF-MR per day. As such, a minimum of two servings per day of DSF-MR is recommended for individuals with obesity (23, 26). Both one and two servings of DSF-MR have been associated with high adherence rates (≥86%) in clinical studies, with adherence of up to 96% observed with two servings per day (24, 26, 35, 36).

In clinical trial methodologies, breakfast is typically selected for MR (26, 37, 38). This approach could foster greater adherence among patients who are initiating a DSF-MR regimen, because lunch or dinner replacement may be relatively constrained by social or work-related norms. Structured patient-led glucose monitoring at initiation of a hypocaloric diet may also enhance adherence by demonstrating the direct glycaemic benefits of the intervention (39).

Lastly, motivational interviewing has been shown to enhance adherence to diabetes care plans, including 1–2 DSF-MRs, by fostering patient engagement and supporting behaviour change in a collaborative manner (24). Demonstrated benefits of this strategy include sustained improvements in HbA1c and weight loss up to 6 months after cessation of holistic interventions, including MNT, exercise prescriptions, and patient education (24). As clinicians may lack the time or experience to deliver motivational interviewing techniques, a suitably qualified coach or therapist may be engaged as part of a multidisciplinary approach to care, discussed further in *3.4 Optimising outcomes with DSF-MR*.

### 3.2 DSF-MR for muscle protection in the era of GLP-1 RAs

GLP-1 RAs are glucose-lowering drugs (GLDs) prescribed for both glycaemic control and weight loss (6). These GLDs can reduce food cravings, enhance satiety, and suppress appetite – a 16–39% reduction in calorie intake has been observed (11, 12). Gastrointestinal adverse effects are also observed, commonly nausea and vomiting with short-acting GLP-1 RAs and diarrhoea with long-acting GLP-1 RAs (40). A key clinical concern is also the loss of lean mass associated with their use, including skeletal muscle mass. For example, a loss of up to 40% lean mass has been reported with semaglutide (8, 9, 13). GLP-1 RAs may also cause vitamin deficiencies through reduction of appetite and food intake. A retrospective analysis found vitamin deficiencies in 12.7% of 461,382 adults prescribed GLP-1 RAs in the previous 6 months, increasing to 22.4% after 12 months of use (10). Vitamin D deficiency was particularly common within this cohort. These findings are clinically significant because individuals with T2DM and overweight/obesity have pre-existing risks of nutritional deficiency and sarcopenia – the progressive loss of skeletal muscle mass, strength, and function – owing to factors such as poor diet and altered nutrient pharmacokinetics (11, 15, 41–49). A low-calorie, high-protein diet, in addition to a tailored resistance exercise program, is therefore recommended to meet macro- and micronutrient requirements and preserve lean mass in individuals who have T2DM and overweight/obesity who are initiated with a GLP-1 RA (4, 12, 50, 51). However, dietary modification may be challenging, owing to reduced appetite (11, 45), gastrointestinal effects (40), and the need to concurrently facilitate glycaemic control and tailor weight loss toward individual patient targets (4).

DSF can help to address this challenge by providing a nutrient-dense, good quality and efficient protein source that can be consumed in small, frequent servings that may be tolerable to those experiencing nausea or loss of appetite with GLP-1 RA use, while also addressing the dietary needs of T2DM and overweight/obesity (14–18, 52). A meal plan that includes DSF-MR, alongside other protein-rich foods or supplements, can also help to preserve muscle in those initiated with a GLP-1 RA (11, 14, 50), and supplement intake of relevant vitamins and minerals. In a 3-month randomised controlled trial, a hypocaloric diet comprising 1–2 DSF-MRs/day was shown to reduce fat mass and visceral adipose tissue versus standard of care, while maintaining fat-free mass; this is suggestive of muscle preservation (26). Approximately ~3.4% of the 117 participants receiving DSF were treated with GLP-1 RAs (26); as such, evidence to support this specific indication for DSF-MR is emerging.

For patients who are diagnosed with sarcopenia or who have experienced rapid and substantial weight loss after 1–2 months of GLP-1 RA initiation, immediate dietary intervention with protein supplementation is recommended (15, 53). The bioactive metabolite beta-hydroxy beta-methylbutyrate (HMB) has also been shown to be effective for improving muscle mass and strength in individuals with sarcopenia (15, 53–57). HMB is a component of selected DSFs, providing further rationale for recommendation of HMB as part of a high-protein diet in this cohort (14–16, 53). Use of HMB is endorsed by several sarcopenia advisory panels and working groups across Asia as part of holistic care that includes structured and individualised muscle-building exercise and nutritional optimisation (58–60).

### 3.3 DSF-MR for diabetes remission

As shown in Table 1, the authors define diabetes remission in alignment with international consensus (4, 61). While not a ‘cure’ for diabetes, diabetes remission indicates near-normalisation of glucose homeostasis and represents a clinically desirable outcome in newly diagnosed T2DM (62).

A diabetes remission program is a structured and holistic framework of intensive behavioural changes, comprising three phases for patients to complete over 1 year. The *TDR* phase is approximately 12 weeks in length and encompasses a diet of 800–1,000 kcal/day, typically achieved through 3–4 liquid MRs (30, 63–65). In the *structured food introduction* phase, individuals adhere to a diet of ~1,200–1,500 kcal/day, for 6–12 weeks, gradually replacing most liquid meals with a food-based diet. This phase should be accompanied by ongoing behavioural modification and physical activity. The final step is the long-term *weight maintenance* phase, which involves 5–6 months of a ~ 1,500–1800 kcal/day diet that may include one serving of liquid MR, with greater calorie restriction if weight rebounds (30, 63–65). Clinical trials that evaluated overall diabetes remission frameworks have reported remission rates of 43–61% at 1 year, associated with 6.8–12 kg weight loss, compared with 4–12% remission rates in control groups. In these studies, 800–860 kcal/day was permitted during TDR, achieved with very low-calorie liquid MRs (28, 63–65).

Emerging real-world evidence indicates that low-calorie diet could also be a viable option for the TDR phase (30, 66). Trenell et al. (30) found that four servings/day of DSF-MR, equating to 900–1,000 kcal/day, resulted in an average 11 kg of weight loss (~11% of body weight) and mean HbA1c reduction of 10.7 mmol/mol (1%) at 12 weeks. Additional benefits included reduction in BMI (−3.7 kg/m^2^) and blood pressure. Similarly, a real-world trial by Valabhji et al. (66), which studied the intake of up to 900 kcal/day of MR products during the TDR phase, reported a mean loss of 15.9 kg (14.4% of body weight) and diabetes remission in one-third of individuals.

It is recommended that MR products selected for TDR provide adequate macro- and micronutrients and fulfill daily protein requirements (4). A high-protein DSF (e.g., 15–18 g/serving) in addition to a low-carbohydrate protein supplement (e.g., soy isolate) may be an effective means to meet total daily protein needs while limiting total energy intake to 800–1,000 kcal/day. Appropriate patient selection for TDR is also crucial, both for efficacy of the program and safety of the individual. Therefore, initiation of a diabetes remission program should be made under medical supervision. Based on diabetes remission study protocols, the typical candidate has T2DM (HbA1c ≥ 6.5%) diagnosed up to 6 years prior, with better results seen in those with a shorter diabetes duration. Candidates are generally overweight or have obesity (30, 63–65), although specific BMI thresholds suitable for TDR initiation may vary by ethnicity. Thresholds to trigger action against T2DM risk have been reported to be higher for those of Caucasian and African descent (≥ 27 kg/m^2^) than those of Chinese or Arab descent (≥ 25 kg/m^2^) (67). Comparatively, the BMI threshold may be as low as 23.9 kg/m^2^ among individuals of South Asian descent (67, 68).

Insulin use is typically an exclusion criterion for TDR (30, 63–65, 69), owing to risk of hypoglycaemia with rapid and significant caloric restriction. However, some patients with T2DM and overweight/obesity receiving insulin may greatly benefit from remission frameworks, and risks may be mitigated through blood glucose monitoring of patients.

Close medical supervision is essential during TDR, given the intensive calorie restriction; weekly follow-ups are recommended, which may later decrease to fortnightly (27). During food reintroduction and weight maintenance phases, 1–3 monthly consultations typically suffice, depending on response (e.g., HbA1c status, maintenance of diabetes remission), patient adherence, and clinician availability (27, 63). After diabetes remission is confirmed, assessment of HbA1c, weight and blood pressure every 3–6 months is appropriate, which may reduce to a yearly assessment if remission is sustained. It is also important to note the impact of ‘metabolic memory’ on assessment needs. Despite remission, individuals may experience persisting effects of previous hyperglycaemia. As such, annual surveillance for complications is recommended, regardless of remission status; this would include foot examination, cardiovascular and kidney health assessment, and neuropathy and retinal screening.

### 3.4 Optimising outcomes with DSF-MR

Adherence to DSF-MR is crucial for optimising outcomes with DSF-MR. A study that compared standard of care against structured Ramadan Nutrition Therapy (sRNT, comprising DSF–partial MR) for 8 weeks found that adherence to DSF was an independent predictor of HbA1c outcome. In this study, adherence was calculated as the proportion of prescribed servings actually consumed, using the formula: *Adherence rate (%) = (number of DSF scoops consumed per day ÷ 7 scoops [1 serving]) × 100%* (70). In the sRNT group, greater adherence to DSF was an independent predictor of HbA1c change (*p* = 0.024), with each 1.0-percentage-point increase in DSF adherence associated with a 0.01-percentage-point reduction in HbA1c (70).

Drawing from clinical experience, the advisory board proposed several strategies to improve real-world DSF-MR adherence among patients. For instance, within a hypocaloric diet conducive to weight loss, pairing DSF with non-starchy and minimally processed vegetables (26, 30, 65, 69) could improve satiety and ongoing adherence to DSF-MR through meal variety. Furthermore, high-fibre vegetable intake can support bowel regularity and increase overall acceptability of DSF as an intervention. Portion size recommendations may be guided by patient hunger and calorie deficit target, or HCPs may refer to the ‘My Healthy Plate’ guide (71), a public health initiative to support individuals in meal planning.

Long-term adherence to DSF-MR may be optimised through coordinated and sustained input from an MDT comprising doctors, dietitians, and diabetes nurse educators. Collaborative care models have proven to improve diabetes management, mood, and psychosocial well-being (4). In Singapore’s Primary Care Network model, a team-based approach enables division of workload and responsibilities among healthcare staff. For instance, a time-constrained physician could have the option to delegate patient queries on meal planning to an allied health colleague who is trained in dietary counselling. The HCP survey indicated that Singaporean GPs rarely engage dietitians in providing MNT. However, the involvement of such specialists is proven to offer metabolic benefits, such as weight loss and glycaemic control, by assisting patients to identify healthier food choices and improve confidence in meal preparation, and by guiding personalised portion control (11). Involvement of a psychologist/behavioural therapist and physiotherapist is also ideal for motivational interviewing and exercise planning, respectively. Additionally, a primary care coordinator may be engaged to function as the anchor of the MDT (4).

Continued MDT support is vital to reinforce patient self-efficacy and to sustain behaviour change (72). To avoid overburdening healthcare staff, digital resources could be used to support clinical care. Telehealth follow-up, remote glucose data monitoring, and shared digital platforms can help to maintain patient contact between appointments with the doctor (4). Additionally, virtual coaching can encourage patient self-management through real-time feedback, while facilitating strategic and timely involvement from the healthcare team (73, 74). A 12-week digitally enabled lifestyle program, in which DSF was used alongside fortnightly virtual coaching sessions and a digital support tool, demonstrated benefits including ~10% weight loss (−11.0 kg), a 1% HbA1c reduction, and improvements in blood pressure (30).

Tailored, patient-centric regimens are essential for optimising outcomes. For example, for individuals initiating a hypocaloric diet, regular monitoring of blood glucose is suggested, as well as ongoing review of GLD regimens (4, 25, 75). Owing to significant improvements in glycaemic control seen with DSF-MR, GLD doses may need to be reduced over time (23, 24).

In some scenarios, pre-emptive reduction of GLDs is advised. An initial ≥50% dose reduction is appropriate to minimise hypoglycaemia risk in patients taking sulfonylureas and meglitinides, adjusted further according to glycaemic response. For those using insulin, an initial 50% dose reduction may be appropriate; however, in patients with markedly elevated HbA1c, a smaller initial reduction (~30%) may be preferred (75). Similarly, pre-emptive tapering of antihypertensives may also be indicated for patients initiating a hypocaloric diet, to reduce the risk of hypotension (25, 76). Evidence indicates that low-calorie diets, including those involving 1 to 3 MRs, can elicit significant reductions in systolic (3–9 mmHg) and diastolic blood pressure (1–6 mmHg) (24, 29, 30, 77). However, blood pressure should be monitored regularly, as patients may require reintroduction of antihypertensive medication (25, 76). Advisor recommendations presented here align with ADA recommendations for treatment deintensification in the setting of “weight loss and/or optimisation of lifestyle behaviours” (4); however, adjustments should ultimately be guided by clinical judgment, as not all patients have typical T2DM physiology.

### 3.5 Treatment algorithm

**Figure 1:**
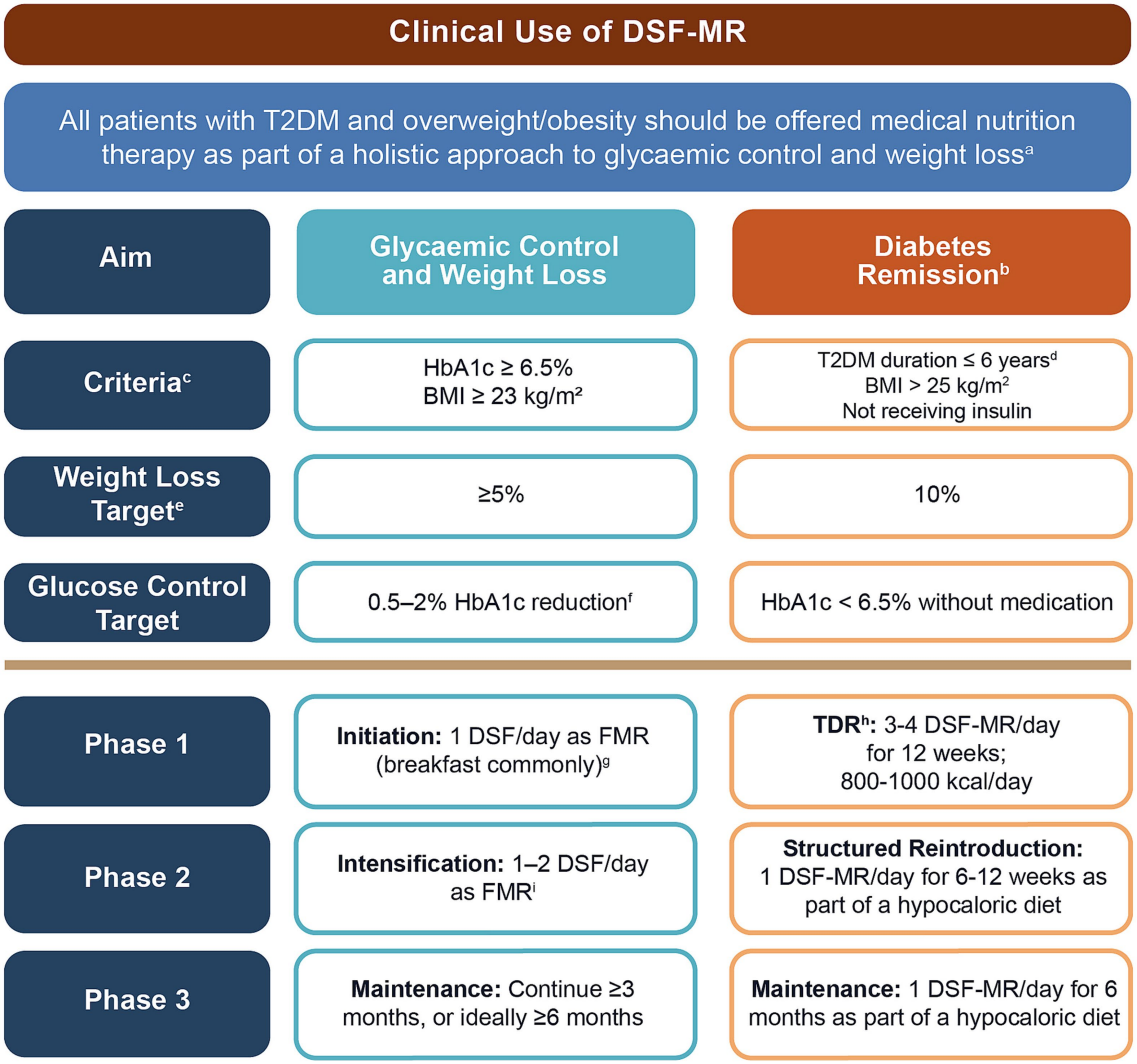
Practical algorithm for integrating DSF-MR into Singaporean diabetes care pathways for individuals with T2DM and overweight/obesity, for the purposes of diabetes control or remission, and weight management (4, 5, 14, 23, 24, 26, 29, 30, 32, 34, 37, 61, 63–65, 67). ^a^ DSF should ideally be used as part of a hypocaloric diet and in conjunction with a structured exercise regimen; benefits may be more pronounced when patient is under the care of a multidisciplinary team and/or counsellors trained in motivational interviewing. DSF-MR can be offered when HbA1c and BMI fall short of clinical goals, particularly for individuals who have had success with MR previously. ^b^ Diabetes remission is defined as HbA1c < 6.5% achieved through intensive behavioural changes and sustained after ≥12 weeks without glucose lowering medications. ^c^ The scope of this treatment algorithm is individuals with T2DM and overweight/obesity (BMI 23.0–32.4 kg/m^2^). BMI thresholds considered suitable to trigger action to address diabetes may vary by ethnicity. ^d^ These criteria are based on pivotal studies on the use of MRs to achieve diabetes remission, which typically permitted individuals with T2DM duration of ≤6 years; however, remission after >6 years of T2DM duration, while less likely, may be achievable and can confer metabolic benefits. ^e^ Weight loss targets for clinically meaningful benefit; additional benefits may be seen with greater weight loss. ^f^ Extent of HbA1c reduction observed may be dependent on baseline value. ^g^ Recommended servings per day should be individualised and adjusted according to BMI at initiation and patient tolerance/motivation. ^h^ Total diet replacement must take place under medical supervision. ^i^ Structured short-term patient-led monitoring at initiation of DSF-MR could be considered to support adherence. BMI, body–mass index; DSF-MR, diabetes-specific formula as meal replacement; FMR, full meal replacement; HbA1c, haemoglobin A1c; T2DM, type 2 diabetes mellitus; TDR, total diet replacement.

To facilitate clinical implementation, an algorithm (Figure 1) was developed to guide the use of DSF-MR in individuals with T2DM and overweight/obesity, targeting glycaemic control, weight loss, or diabetes remission. The algorithm integrates expert consensus, existing guideline recommendations, and pivotal trial protocols.

## 4 Discussion

Overall, the literature review demonstrated that DSF-MRs are a viable and versatile dietary intervention that may be integrated across the continuum of T2DM, as part of a holistic approach to care, to achieve glycaemic control, weight loss targets, and ultimately optimise cardiometabolic health (24, 26, 29, 30, 62, 64). Even so, the Singaporean HCP survey indicated that uptake of DSF may be limited by its perceived costliness. It is important to note that the cost of a DSF-MR serving [typically $3–4 SGD ($2–3 USD)] (78, 79) is likely lower than a typical takeout or restaurant meal expense. Moreover, DSF-MR is considerably more cost-effective, given that it is more nutrient-dense, complete, satiating, and tailored to manage T2DM and overweight/obesity.

In addition to correcting misconceptions of cost, HCPs could emphasise the value of DSF-MR when counselling patients, and consider tailoring this discussion to the individual to encourage uptake. Some patients may be receptive to learning about the demonstrated metabolic benefits, while other patients may prefer to understand the potential healthcare cost savings associated with DSF. For example, a 65.6% healthcare cost reduction has been reported for older patients with protein-caloric malnutrition and T2DM who received DSF, owing to fewer emergency centre visits, hospital admissions, and days spent in hospital (80). Another study found significant reductions in mortality, insulin use, and intensive care unit usage costs in patients receiving DSF compared with standard formulas (81). As a final point, our author group encourages HCPs to highlight to patients that MNT and pharmacological therapy are critical and complementary components of holistic T2DM and obesity management, which may help to optimise acceptance of DSF-MR.

Recommendations presented here build on existing recommendations published in a 2024 joint statement by the Australian Diabetes Society, Australian Diabetes Educators Association, Royal Australian College of General Practitioners, and Dietitians Australia (14). Further studies on DSF-MR applications in the past year, particularly for muscle protection, warranted updates to these existing recommendations. Furthermore, the relatively prescriptive recommendations presented here could be of greater relevance to the Singaporean setting, where DSF uptake has been shown to be suboptimal. Adoption of these recommendations may be assessed through quality improvement initiatives or clinical practice audits.

This multicomponent initiative had several strengths. First, we performed an in-depth and specialised survey questions targeted at GPs, dietitians, and endocrinologists. The response rate was sufficient to identify current trends and gaps in Singaporean MNT practice, including clinician uncertainty and inexperience. Second, the draft recommendation statements were the culmination of clinical experience and a comprehensive literature review whereby most papers identified were rated as high-grade evidence per the ADA grading framework. The consensus recommendations were refined by advisors through a modified Delphi consensus process. A complete (100%) response from all advisors was received in both rounds of online voting, which was blinded and independent to minimise bias. All consensus recommendations were subjected to two rounds of voting, even if consensus was achieved on any statement after the first round, to strengthen the reliability and robustness of the final recommendation. Another key strength of this consensus recommendations is the inclusion of a simplified treatment algorithm that can facilitate a structured, stepwise approach to implementing DSF-MR recommendations in practice, addressing the time and confidence limitations faced by many HCPs.

Some limitations should also be acknowledged. Several gaps in the literature were noted, which are bridged in our recommendations with clinical experience, real-world evidence and expert hypotheses where feasible. For instance, expert recommendations for targeting diabetes remission are based on robust evidence from total diet replacement frameworks using MNT, as well as specific real-world evidence for DSF-MR (30, 63–65, 69). Clinical studies on DSF-MR for diabetes remission are currently underway (82, 83), and the outcomes may optimise or strengthen the recommendations presented here. Similarly, emerging evidence indicates that DSF-MR has potential for muscle protection, which has translated into two practical recommendations presented here. However, there are insufficient trial protocols on this application of DSF that are currently available for adaptation into a treatment algorithm. A formal systematic review of relevant databases was beyond the scope of this manuscript, but it may have identified additional publications to help address these gaps. Similarly, there is currently limited evidence to quantify the interaction between GLP-1 RAs and DSF-MRs. Some studies indicate that DSFs may modulate endogenous GLP-1 secretion more effectively than standard meals and formulas (84–86). As such, DSF-MR has the potential to mitigate the rebound weight gain characteristic of GLP-1 RA treatment cessation if a synergistic effect between these two interventions can be demonstrated. Further studies are needed to clarify whether any synergistic or substitution effects exist in this context, and refinement of these expert recommendations and the algorithm are indicated as additional literature becomes available.

## 5 Conclusion

DSF-MR is an evidence-based structured MNT for individuals with T2DM and overweight/obesity who require nutritional support to meet their clinical targets. This consensus addressed key gaps in real-world adoption of DSF-MR through a rigorous narrative literature review and consensus process with an expert multidisciplinary panel. The consensus recommendations offer practical and detailed guidance for addressing four key clinical needs: improving glycaemic control, supporting diabetes remission, preserving muscle mass, and optimising outcomes. Overall, these recommendations strengthen the evidence base for DSF-MR and support its patient-centred integration into diabetes care in Singapore.

## Data Availability

The original contributions presented in the study are included in the article/Supplementary material, further inquiries can be directed to the corresponding author.
