# Supplementary material for: Diabetes-specific formula as meal replacement for individuals with type 2 diabetes mellitus and overweight or obesity: a Singapore expert consensus
Source: Front Nutr. 2026 May 13;13:1810277. doi: 10.3389/fnut.2026.1810277 (PMC13214565; doi:10.3389/fnut.2026.1810277)
Supplement: Supplementary file 1 [file Data_Sheet_1.docx]

**Supplementary Information 1.** Healthcare professional (HCP) survey: Medical nutrition therapy for patients with type 2 diabetes mellitus and overweight/obesity

**A. HCP Background Questions**

*Instruction to respondents: The following questions relate to your role as a healthcare provider and the types of patients you treat.*

1. What best describes your primary healthcare role?
   1. General practitioner
   2. Dietician
   3. Specialist
   4. Other (please specify)
2. Please specify the primary locations for your practice [*multiple selections allowed*]
   1. Private clinic
   2. Polyclinic
   3. Public hospital
   4. Private hospital
3. Approximately what proportion of your patients have haemoglobin A1c ≥7%?
   1. <25%
   2. 26–50%
   3. 51–75%
   4. >75%

*Instructions to respondents:From this point forward, questions will* *focus on patients who have type 2 diabetes mellitus and overweight/mild obesity, as profiled below. Please* ***omit from your responses*** *your approach to patients with higher grades of obesity (i.e., those requiring surgical intervention).*

Type 2 diabetes mellitus: Fasting plasma glucose ≥126 mg/dL
Oral glucose tolerance test plasma glucose ≥200 mg/dL
Random plasma glucose ≥200 mg/dL
Haemoglobin A1C ≥6.5%

Overweight: 23.0 ≤ BMI ≤ 27.4
Obese I (mild): 27.5 ≤ BMI ≤ 32.4
Do not consider: BMI ≥ 32.5

**B. Attitude Toward Nutritional Therapy**

1. Please rank the following interventions in terms of importance when managing patients with type 2 diabetes and overweight/obesity *(choose only one option for each row)*.

|  | **Diet Modification** | **Physical Activity** | **Behavioural Therapy** | **Pharmacology** |
| --- | --- | --- | --- | --- |
| **Most important (1)** |  |  |  |  |
| **(2)** |  |  |  |  |
| **(3)** |  |  |  |  |
| **Least important (4)** |  |  |  |  |

Providing nutritional advice to patients

1. I have **adequate training and resources** to provide detailed nutritional advice to patients with type 2 diabetes and overweight/obesity
   1. Strongly agree
   2. Agree
   3. Disagree
   4. Strongly disagree
2. I have **adequate time** to provide detailed nutritional advice to patients with type 2 diabetes and overweight/obesity
   1. Strongly agree
   2. Agree
   3. Disagree
   4. Strongly disagree

**C. Dietary Recommendations**

1. When counselling patients with type 2 Diabetes and overweight/obesity, what is the **most common dietary recommendation** you offer to your patients? [*Open text*]
2. Approximately what proportion of your patients achieved **their glucose control goal** after commencing this dietary recommendation?
   1. <25%
   2. 26–50%
   3. 51–75%
   4. >75%
3. What is **the biggest challenge you or your patient experiences** in relation to implementing this dietary recommendation? [*Open text*]
4. Do you recommend, prescribe, or provide **diabetes-specific formula meal replacements** (DSF-MR) to patients with type 2 diabetes and overweight/obesity?
   1. Yes (go to **section D**)
   2. No (go to **section E**)

**D. DSF-MR Recommended (only presented if answer to Q8 is *yes*)**

**These questions are for those who recommend/prescribe/provide DSF-MR.**

1. I am **confident** providing condition-specific formulations for dietary interventions with
   type 2 diabetes and overweight/obesity

- Yes
- No

1. If yes, **what is your confidence level** for providing condition-specific formulations for dietary interventions with type 2 diabetes and overweight/obesity?
2. Very high confidence
3. High confidence
4. Low confidence
5. At what **time point** do you recommend diabetes-specific formulations as meal replacements to patients with type 2 diabetes mellitus and overweight/obesity?
6. As soon as possible (initial diagnosis and assessment)
7. After short-term follow-up (1–3 months)
8. After mid-term follow-up (6–12 months)
9. During long-term follow-up (>12 months)
10. For which **types of patients** would you recommend diabetes-specific formulations as meal replacements? Please select all that apply [*multiple selections allowed*]

- Diagnosis

1. Having diagnosis of type 2 Diabetes
2. Being overweight
3. Being obese (obesity stage 1)

- Psychosocial factors

1. Patients with medication adherence issues
2. Patients of medium socioeconomic class
3. Patients of high socioeconomic class

- Others (please specify) [*Open text box*]

1. **Why** do you recommend, prescribe, or provide diabetes-specific formulations as meal replacements? [*Open text box*]
2. Studies have shown that diabetes-specific formulations, when given as meal replacements, improve glycaemic control, lead to diabetes remission, improve satiety, induce weight loss, reduce visceral fat, improve cardiometabolic risk factors, and reduce overall healthcare cost (Noronha et al, 2022; Mechanick et al., 2020; Trenell et al., 2024; Bynoe et al., 2020; Hocking et al., 2024).
    **What specific benefits,** if any, **have you observed** in your patients who use diabetes-specific formulations as meal replacements? [*Open text box*]
3. Do you face any of the following **challenges** when recommending, prescribing, or providing diabetes-specific formulations as meal replacements? [*Multiple selections allowed*]

- Lack of patient acceptance (e.g., patient perceives it to be costly or ineffective)
- Lack of patient compliance with treatment plan
- Your awareness of available products is limited
- Other challenges (please specify)
- I do not face any challenges

**E. DSF-MR Not Recommended (only presented if answer to Q8 is *no*)**

**These questions are only for those who do not recommend/prescribe/provide DSF-MR.**

1. Please specify what barriers are most relevant to **your decision not to recommend, prescribe, or provide** diabetes-specific formulations as meal replacements to patients with type 2 diabetes and overweight/obesity.
   1. Your awareness of available products is limited
   2. You perceive it to be costly
   3. You perceive the product to have limited effectiveness
   4. You perceive the product to result in nutritional imbalance
   5. Your patient does not accept this intervention (e.g., perceives it as costly, inconvenient, or ineffective)
   6. Other (please specify)
2. What factors **may encourage you** to recommend, prescribe, or provide diabetes-specific formulations to patients with type 2 diabetes and overweight/obesity?
3. Evidence-based recommendations for DSF
4. Improved patient education and engagement material
5. Cost-effectiveness data on improving glycaemic and weight control
6. Other (please specify)

**Supplementary Information 2.** Key results from the healthcare professional (HCP) survey


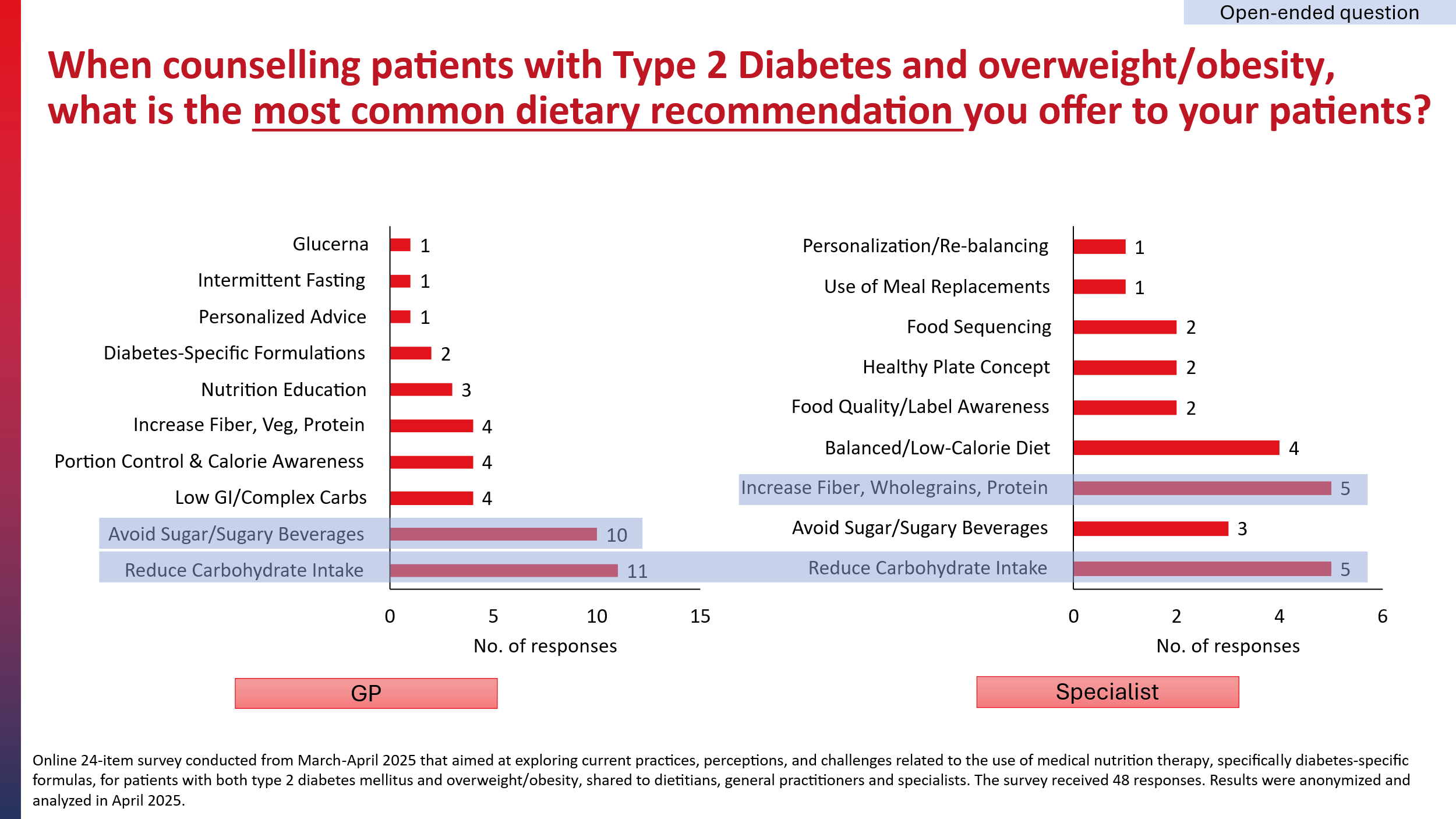


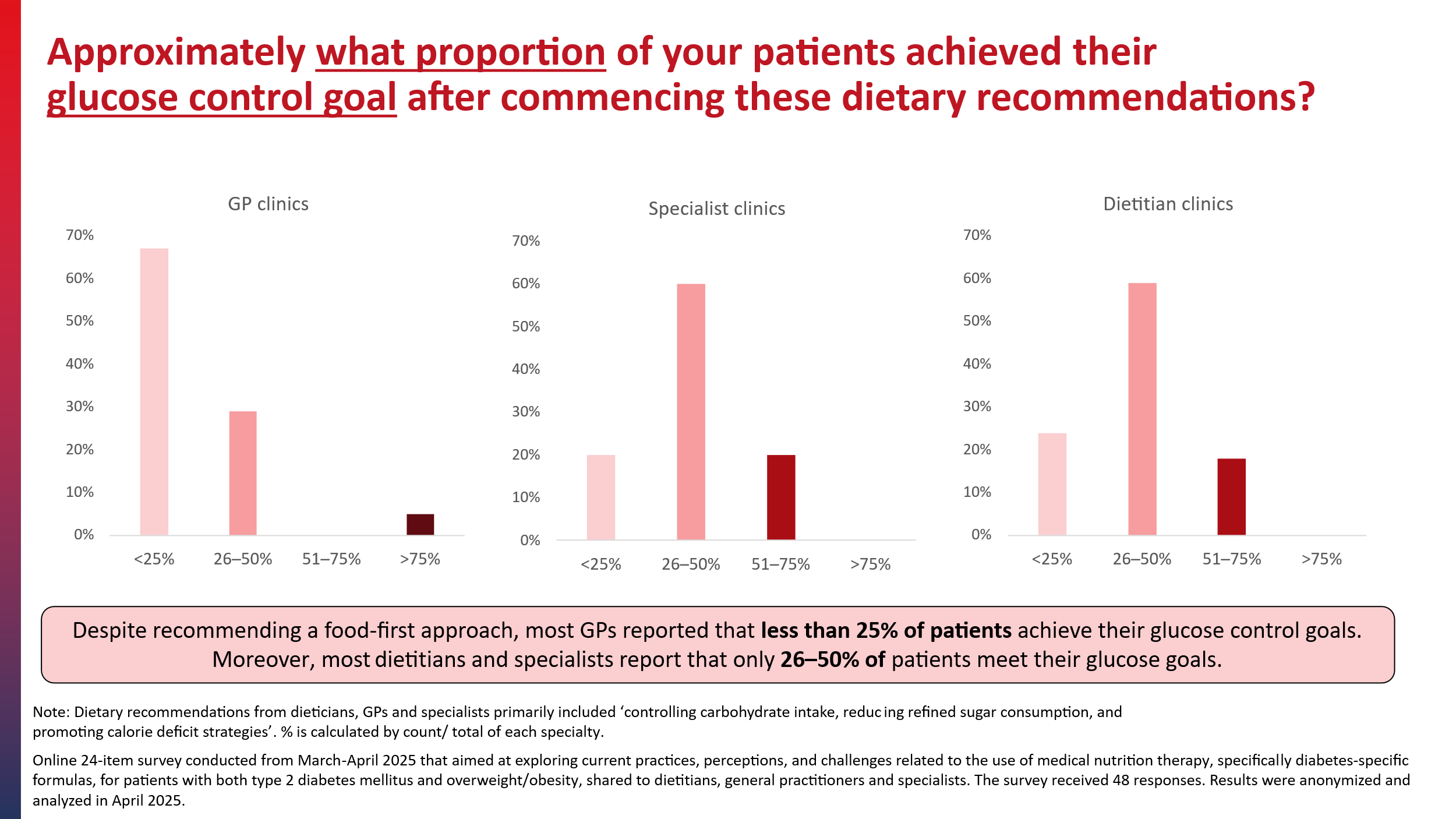


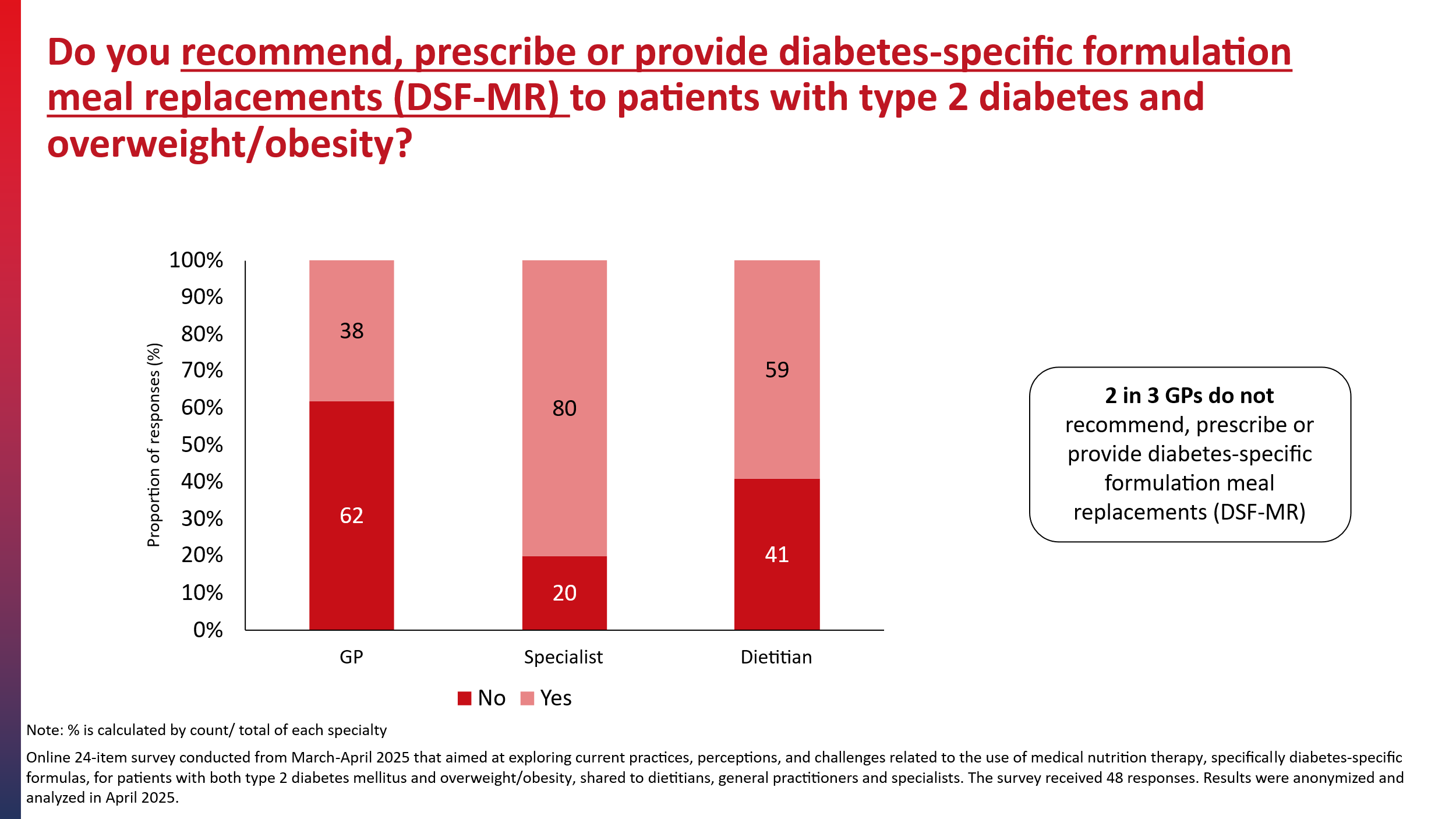


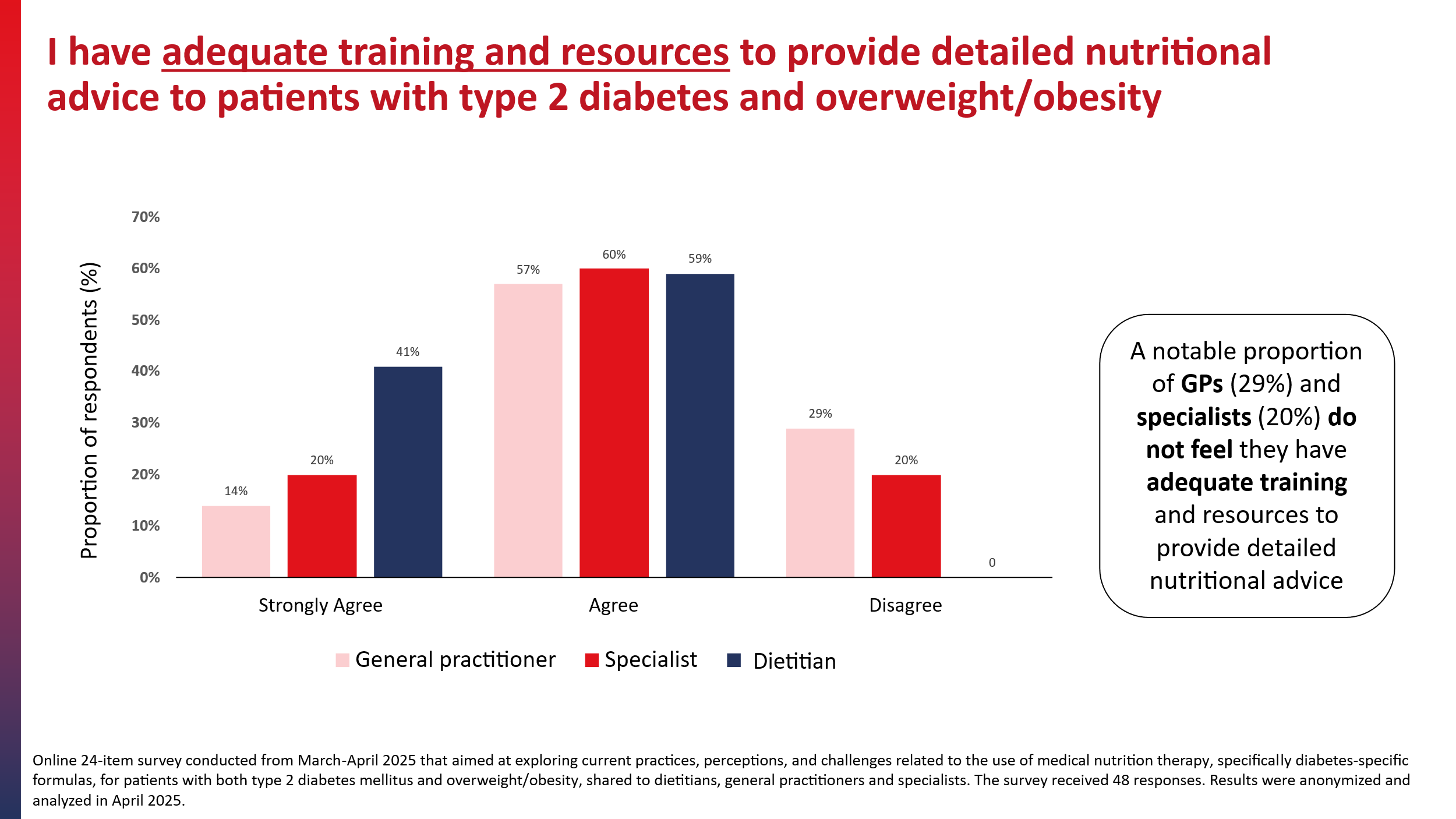

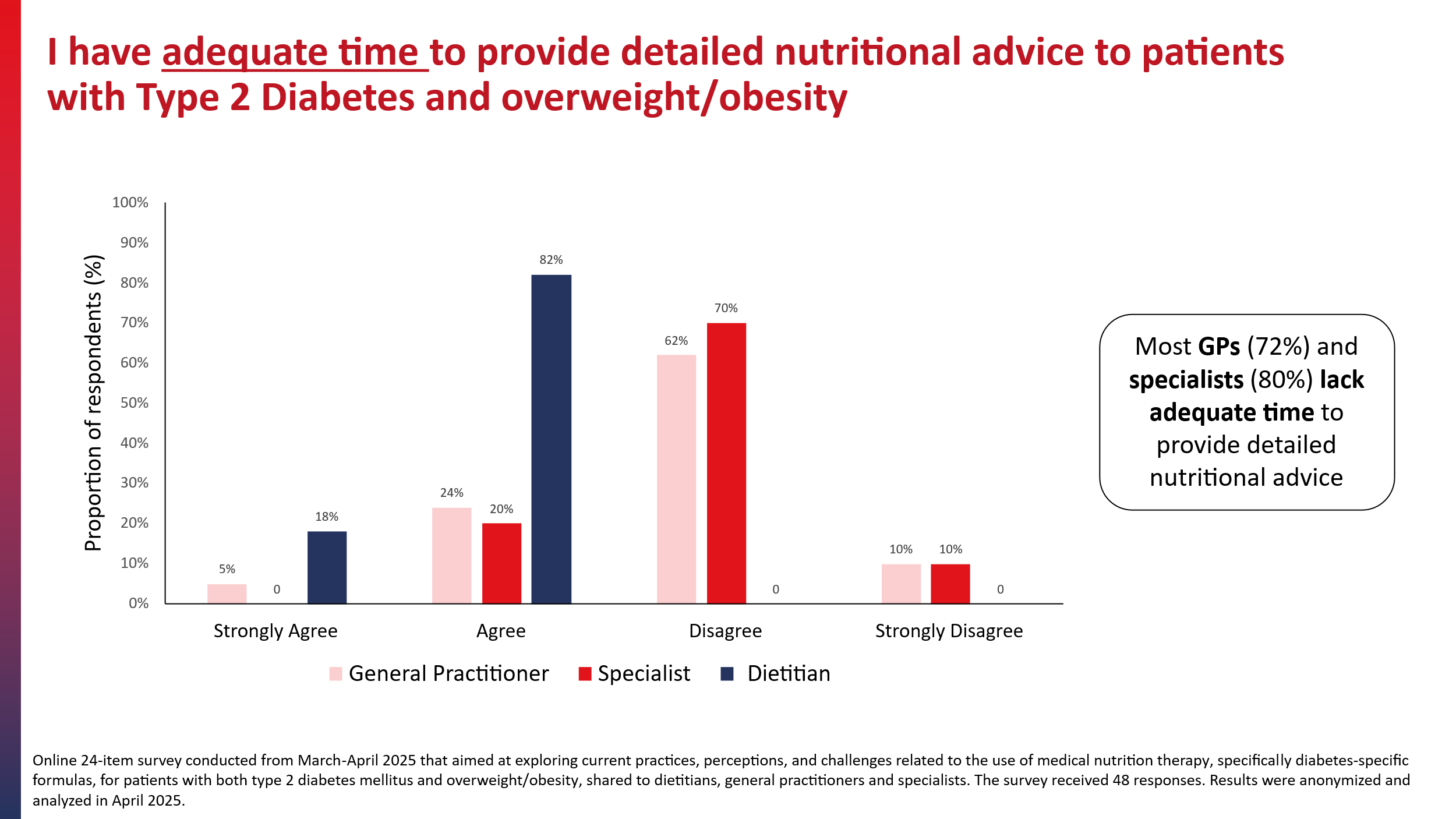


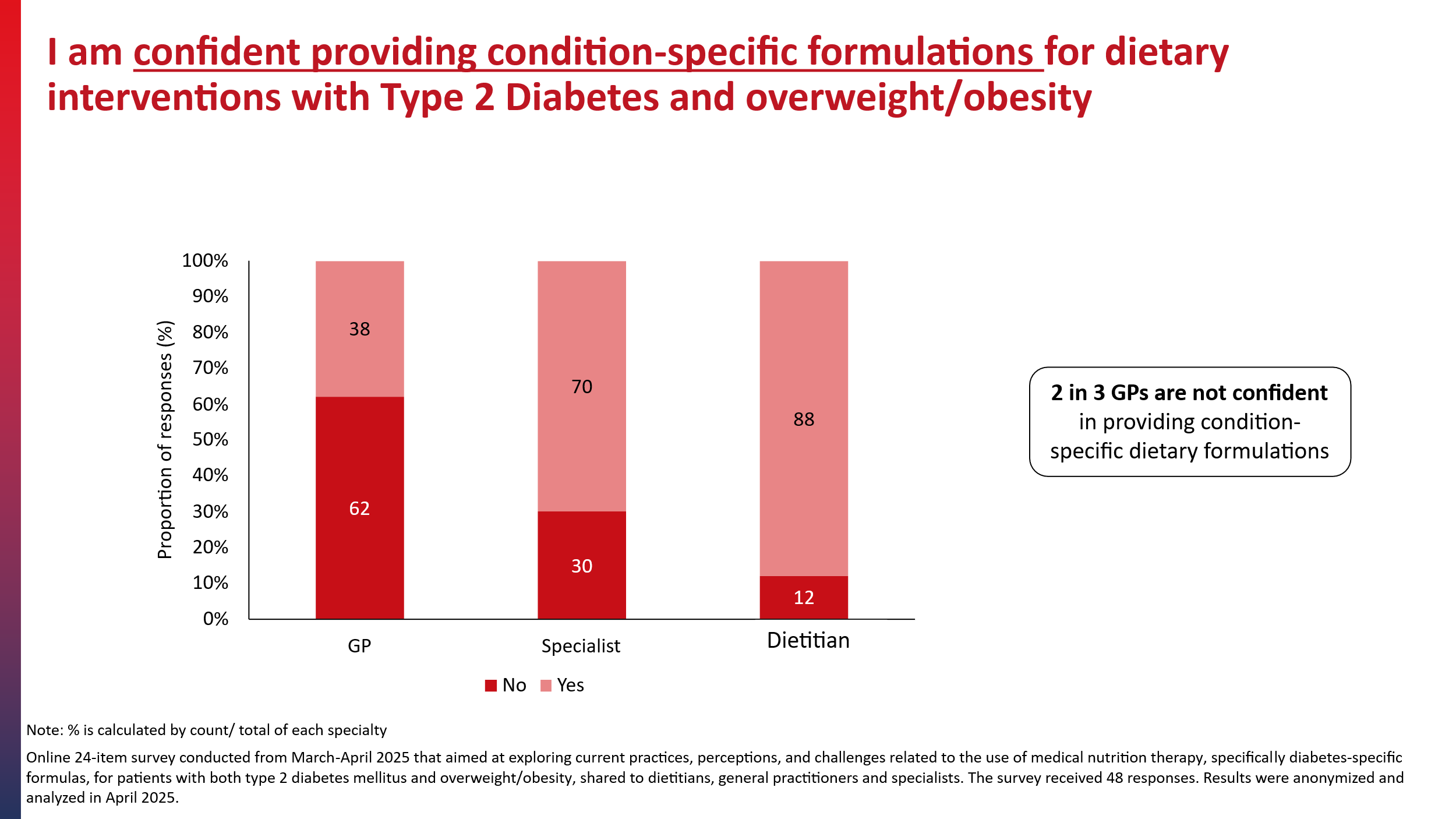


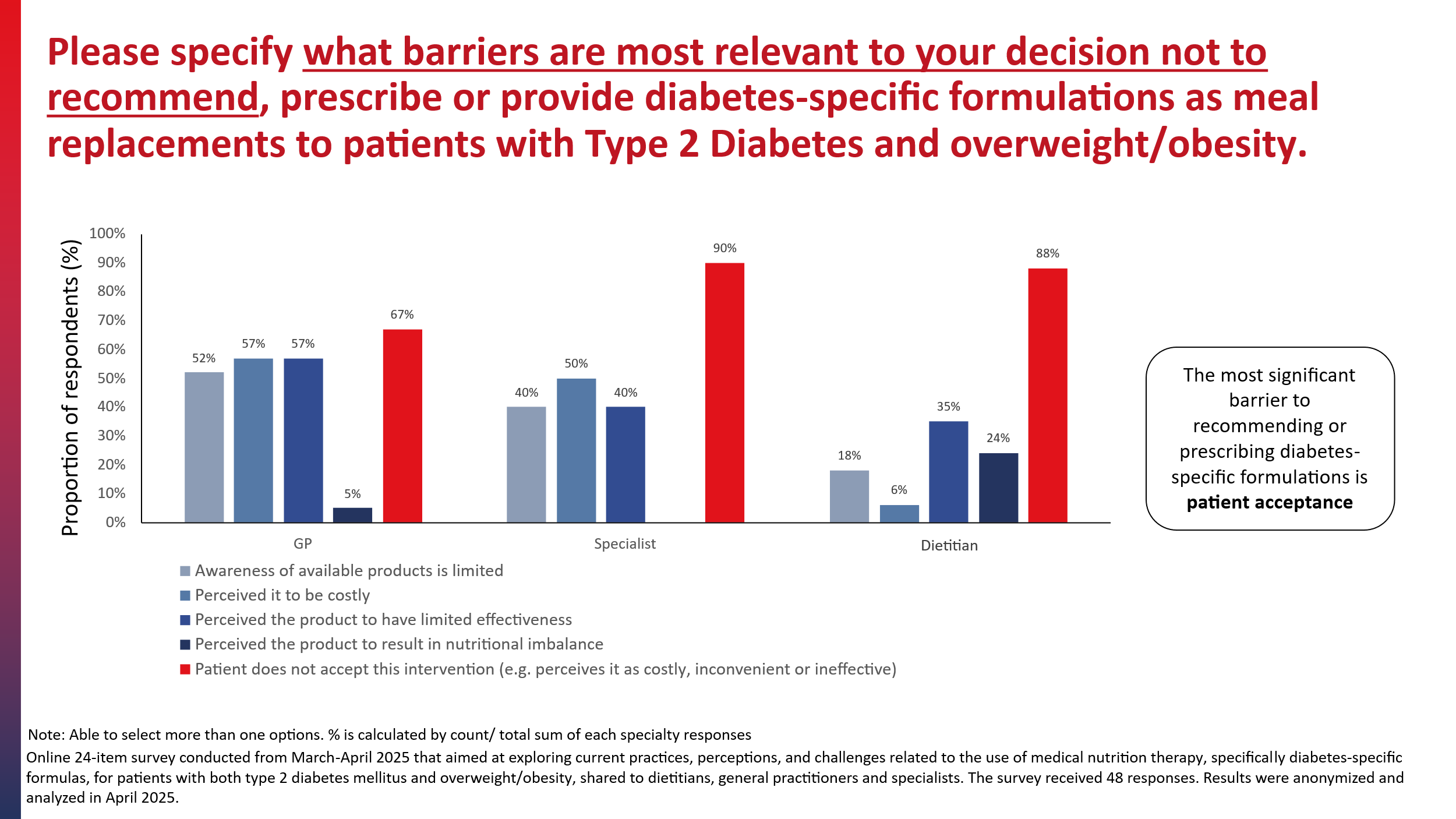


Results presented here are pertinent to the paper. Please contact the corresponding author for a copy of complete survey results.

**Supplementary Information 3.** Evolution of expert recommendations between each Delphi voting round.

| **Topic** | **No.** | **Draft statement** | **Round 1 voting agreement** | **Revised statements^a^** | **Round 2 voting agreement** | **Final statements^a^** | **Corrected voting results^b^** |
| --- | --- | --- | --- | --- | --- | --- | --- |
| DSF-MR for patients with T2DM and overweight/obesity for better glucose control | 1.1 | As part of a standard approach to medical nutrition therapy for diabetes care, diabetes-specific formula (DSF) should be recommended as full meal replacement (FMR) to all patients with type 2 diabetes mellitus (T2DM) and overweight/obesity, particularly those who:  • Are not meeting glycaemic targets, weight loss targets, or nutritional needs with standard dietary approaches  • Are willing to initiate and sustain DSF-MR for the duration required to achieve the clinical goals set by the healthcare professional  • Are unable to comply with a food-first approach or previously recommended meal plans  • Have a busy lifestyle and value convenient and fast meals  • Are not willing to increase pill burden for glycaemic control  • Have health conditions that may limit physical activity | 63.6% | As part of a standard approach to medical nutrition therapy for diabetes care, diabetes-specific formula (DSF) should be recommended as full meal replacement (FMR) to ~~all~~ patients with both type 2 diabetes mellitus (T2DM) and overweight/obesity, particularly those who: • Are not meeting glycaemic or weight loss targets with previous dietary interventions; those who are reluctant to increase their medication burden for glycaemic control; or those who have health conditions that limit physical activity, making dietary intervention a more central treatment strategy  • Are unable to comply with a whole food-based approach or previously recommended meal plans, perhaps owing to limited resources, low confidence, limited nutrition literacy or cooking skills; or those who have a busy lifestyle and value convenience  • Are willing to initiate and sustain DSF-MR for the duration required to achieve the clinical goals set by the healthcare professional | 72.7% | No further change made | 72.7% |
|  | 1.2 | DSF should be used as a full replacement for 1–2 meals per day for ≥3 months in patients with T2DM and overweight/obesity, and as long as required to achieve clinical goals, and may have a greater effect on HbA1c and bodyweight when used for ≥6 months. | 63.6% | DSF can be used as a full replacement for 1–2 meals per day for ≥3 months in patients with T2DM and overweight/obesity, and as long as required to achieve clinical goals, and may have a greater effect on HbA1c and bodyweight when used for ≥6 months.  DSF should ideally be used as part of a hypocaloric diet and in conjunction with a structured exercise regimen. | 90.9% | No further change made | 90.9% |
|  | 1.3 | The benefits of a standard approach to medical nutrition therapy for glycaemic control and weight loss – which includes DSF as meal replacement for 1–2 meals/day – may be optimized and maintained if recommended alongside motivational interviewing. | 72.7% | The benefits of a structured lifestyle intervention for glycaemic control and weight loss – which includes DSF as meal replacement for 1–2 meals/day within a hypocaloric diet – may be more pronounced if recommended alongside motivational interviewing. | 100.0% | No further change made | 100.0% |
|  | 1.4 | Adherence to meal replacement with DSF as part of a hypocaloric diet may be optimized by starting with modest dietary changes (1 meal replacement/day) and gradually intensifying the number of meal replacements to achieve the recommended servings/day for clinically recommended weight loss or glycaemic goals.  Recommended servings per day should be individualized and sustained according to body mass index at initiation, and glycaemic and weight loss targets. | 63.6% | To optimize adherence to meal replacement with DSF, as part of a hypocaloric diet, a stepwise approach is recommended. Start with 1 meal replacement/day and gradually increase the number of meal replacements/day, to achieve and sustain the recommended number of servings/day indicated for weight loss or glycaemic goals. Recommended servings per day should be individualized and adjusted according to body mass index at initiation and patient tolerance/motivation. | 81.8% | To optimise adherence to meal replacement with DSF, as part of a hypocaloric diet, a stepwise approach is recommended. Start with 1 meal replacement/day and gradually increase the number of meal replacements/day, to achieve and sustain the recommended number of servings/day indicated for weight loss or glycaemic goals. Recommended servings per day should be individualised and adjusted according to body mass index at initiation and patient tolerance/motivation. | 90.9% |
|  | 1.5 | At initiation, DSF as a replacement for breakfast is most studied and could be more acceptable and convenient to patients than replacing other meals.  Meal/s to be replaced with DSF can also be determined by post-prandial glycaemic profiles. | 90.9% | At initiation, DSF as a replacement for breakfast is most studied and could be more acceptable and convenient to patients than replacing other meals. Meal/s to be replaced with DSF can also be determined by post-prandial glycaemic profiles, prioritizing the meal that results in the largest glucose excursion. | 81.8% | At initiation, DSF as a replacement for breakfast is most studied and could be more acceptable and convenient to patients than replacing other meals. Meal/s to be replaced with DSF can also be determined by post-prandial glycaemic profiles, prioritising the meal that results in the largest glucose excursion. | 90.9% |
|  | 1.6 | Short-term patient-led glucose monitoring at initiation of DSF-MR could be considered to help patients visualise improvements in glycaemic control and encourage compliance with DSF as a meal replacement. | 63.6% | Structured short-term patient-led glucose monitoring at initiation of DSF-MR (e.g., tracking blood glucose levels before and after meals), along with maintaining a food diary, could be considered to help patients visualise improvements in glycaemic control and encourage compliance with DSF as a meal replacement. **Disclaimer:** The use of DSF-MR as full replacement of 1–2 meals per day does not require monitoring for safety purposes, as changes will be gradual.  Intensive and/or ongoing monitoring may be indicated for other aspects of patient care, such as medication adjustment and hypoglycemia prevention. | 54.5% | Structured short-term patient-led glucose monitoring at initiation of DSF-MR (e.g., tracking blood glucose levels before and after meals), along with maintaining a food diary, could be considered to help patients visualise improvements in glycaemic control and encourage compliance with DSF as a meal replacement.  **Disclaimer:** • The use of DSF-MR as full replacement of 1–2 meals per day does not require monitoring for safety purposes, as changes will be gradual.  • Intensive and/or ongoing monitoring may be indicated for other aspects of patient care, such as medication adjustment and hypoglycaemia prevention. | 72.7% |
| DSF-MR for patients with T2DM and overweight/obesity for muscle protection in the era of new drugs (e.g., GLP-1 receptor agonists [RAs]) | 2.1 | Patients with T2DM and overweight/obesity who are receiving GLP-1 RAs can benefit from nutritionally dense, complete, and balanced hypocaloric meals to address the need for glycaemic control, weight loss, and adequate protein for muscle protection while accounting for limited appetite and/or poor dietary habits that can result in nutrient deficiencies. | 90.9% | No further change made | 90.9% | No further change made | 90.9% |
|  | 2.2 | At GLP1-RA treatment initiation, a protein intake goal of 1.2–1.5 g/kg/day of high biological value could be achieved with a meal plan that includes DSF as meal replacement for 1–2 meals a day, in addition to protein-rich foods or protein modular supplements.  This meal plan will provide a nutritionally dense, complete, and balanced hypocaloric diet to support muscle health and glycaemic control while reducing overall body weight in patients with T2DM and overweight/obesity. | 81.8% | No further change made | 100.0% | No further change made | 100.0% |
|  | 2.3 | For patients who are diagnosed with sarcopenia or have experienced rapid weight loss following GLP-1 RA initiation, a protein intake goal of 1.2–1.5 g/kg/day of high biological value could be achieved with a meal plan that includes DSF as meal replacement for 1–2 meals a day, in addition to protein-rich foods or protein modular supplements.  This meal plan could provide a nutritionally dense, complete, and balanced hypocaloric diet to restore muscle health in combination with strength training, achieve glycaemic control, and reduce overall body weight in patients with T2DM and overweight/obesity. | 81.8% | For patients with T2DM and overweight/obesity who are diagnosed with sarcopenia or have experienced rapid weight loss following GLP-1 RA initiation, a protein intake goal of 1.2–1.5 g/kg/day of high biological value could be achieved with a meal plan that includes DSF as meal replacement for 1–2 meals a day, in addition to protein-rich foods or protein modular supplements. This meal plan could provide a nutritionally dense, complete, and balanced hypocaloric diet to restore muscle health (in combination with strength training), improve glycaemic control, and facilitate continued weight loss if clinically indicated. | 90.9% | No further change made | 90.9% |
| DSF-MR for patients with overweight/obesity and recently diagnosed T2DM to achieve diabetes remission | 3.1 | A diet of 800–1000 kcal/day for 12 weeks, which could be achieved by total diet replacement (TDR) with DSF, can help to attain diabetes remission in patients with overweight/obesity and newly diagnosed T2DM (≤6 years).  Diabetes remission is defined as HbA1c <6.5% achieved through intensive behavioural changes and sustained after ≥12 weeks without glucose lowering medications.  Disclaimer:  • BMI thresholds considered suitable for TDR initiation may vary by ethnicity. | 63.6% | A diet of 800–1000 kcal/day for 12 weeks, which could be achieved through total diet replacement (TDR) with DSF, can help to attain diabetes remission in patients with overweight/obesity and newly diagnosed T2DM (≤6 years).  Diabetes remission is defined as HbA1c <6.5% achieved through intensive behavioural changes and sustained after ≥12 weeks without glucose lowering medications.  **Disclaimer:**  • Total diet replacement must take place under medical supervision • Patients who are receiving insulin are not suitable candidates for total diet replacement • BMI thresholds considered suitable for TDR initiation may vary by ethnicity. | 63.6% | A diet of 800–1000 kcal/day for 12 weeks, which could be achieved through TDR with DSF, can help to attain diabetes remission in patients with overweight/obesity and newly diagnosed T2DM (≤6 years).  Diabetes remission is defined as HbA1c <6.5% achieved through intensive behavioural changes and sustained after ≥12 weeks without glucose lowering medications.  Disclaimer:  • TDR must take place under medical supervision • Patients who are receiving insulin are not suitable candidates for TDR • BMI thresholds considered suitable for TDR initiation may vary by ethnicity. | 72.7% |
|  | 3.2 | A 6- to 12-week structured food reintroduction phase, which includes DSF to replace 1 meal a day within a hypocaloric diet, can support glycaemic and weight control after the TDR phase. | 81.8% | No further change made | 90.9% | No further change made | 90.9% |
|  | 3.3 | A 6-month weight maintenance phase, which may include DSF to replace 1 meal a day within a hypocaloric diet, could help to support glycaemic and weight control after the structured food introduction phase. | 90.9% | No further change made | 90.9% | No further change made | 90.9% |
| Optimising outcomes for patients with T2DM and overweight/obesity using DSF-MR for glycaemic control and weight loss | 4.1 | Within a hypocaloric diet or total diet replacement framework, DSF could be accompanied by a portion of non-starchy high-fibre vegetables to improve patient satisfaction. | 72.7% | Within a hypocaloric diet or TDR framework, DSF could be accompanied by a portion of non-starchy high-fibre vegetables to support satiety, enhance tolerability of the diet, and improve overall patient satisfaction. | 81.8% | No further change made | 81.8% |
|  | 4.2 | Adherence to a hypocaloric diet, which includes DSF-MR for glycaemic control and weight loss, could be optimized by routine, coordinated, and sustained involvement of a multidisciplinary team (MDT) that includes a doctor, dietitian, physiotherapist, and diabetes nurse educator.  MDT members must provide specialised advice and share responsibility for monitoring patient progress. | 63.6% | Adherence to a hypocaloric diet, which includes DSF-MR for glycaemic control and weight loss, can be optimized by routine, coordinated, and sustained involvement of a multidisciplinary team (MDT) that includes a doctor, dietitian, physiotherapist, and diabetes nurse educator. MDT members must provide specialised advice and share responsibility for monitoring patient progress. | 72.7% | Adherence to a hypocaloric diet, which includes DSF-MR for glycaemic control and weight loss, can be optimised by routine, coordinated, and sustained involvement of a multidisciplinary team (MDT) that includes a doctor, dietitian, physiotherapist, and diabetes nurse educator. MDT members must provide specialised advice and share responsibility for monitoring patient progress. | 81.8% |
|  | 4.3 | For patients following a hypocaloric diet, which includes DSF-MR for glycaemic control and weight loss, remote check-ins, combining in-person visits with telehealth, staged follow-up, and shared digital platforms could help to maintain frequent touchpoints with patients without overburdening staff or healthcare resources. | 81.8% | For patients following a hypocaloric diet, which includes DSF-MR for glycaemic control and weight loss, remote check-ins, combining in-person visits with telehealth, staged follow-up, and shared digital platforms can be utilised to maintain frequent touchpoints with patients without overburdening staff or healthcare resources. | 100.0% | No further change made | 100.0% |
|  | 4.4 | Pre-emptive tapering of insulin or sulfonylureas is recommended for patients who intend to follow a hypocaloric diet. The tapering schedule should be tailored to the individual patient’s medical condition and guided by clinical judgement. | 63.6% | Pre-emptive tapering of insulin or sulfonylureas is recommended for patients who intend to follow a hypocaloric diet. The tapering schedule should be tailored to the individual patient’s medical condition and guided by clinical judgement.  Disclaimer:  • Factors that may influence the tapering schedule include assessment of hypoglycemia risk, baseline HbA1c, and meal replacement plan.  • Adjustments to the treatment plan should be made through shared decision-making and guided by close blood glucose monitoring. | 63.6% | Pre-emptive tapering of insulin or sulfonylureas is recommended for patients who intend to follow a hypocaloric diet. The tapering schedule should be tailored to the individual patient’s medical condition and guided by clinical judgement.  Disclaimer:  • Factors that may influence the tapering schedule include assessment of hypoglycaemia risk, baseline HbA1c, and meal replacement plan.  • Adjustments to the treatment plan should be made through shared decision-making and guided by close blood glucose monitoring. | 81.8% |
|  | 4.5 | Pre-emptive tapering of antihypertensives may be considered for patients who intend to follow a hypocaloric diet. The tapering schedule should be tailored to the individual patient’s clinical condition, specific antihypertensive prescribed, and response to hypocaloric diet. | 72.7% | Pre-emptive tapering of antihypertensives may be considered for patients who intend to follow a hypocaloric diet. The tapering schedule should be tailored to the individual patient’s clinical condition (e.g., through home blood pressure readings), specific antihypertensive prescribed, and response to hypocaloric diet (e.g., degree of weight loss). | 81.8% | Pre-emptive tapering of antihypertensive medications may be considered for patients who intend to follow a hypocaloric diet. The tapering schedule should be tailored to the individual patient’s clinical condition (e.g., through home blood pressure readings), specific antihypertensive medication prescribed, and response to hypocaloric diet (e.g., degree of weight loss). | 90.9% |
|  | 4.6 | Regular review of glucose-lowering medication dosage is advised for all patients with T2DM and overweight/obesity who have initiated a hypocaloric diet. | 100.0% | No further change made | 100.0% | No further change made | 100.0% |

^a^ Changes marked in red.
^b^ Several comments received at round 2 voting were to request use of British English rather than US English, given the Singapore focus of the manuscript; as such, the relevant votes of ‘Accepted with Revisions’ were adjusted to ‘Accept’ upon language modification, and the final proportion of agreement was calculated accordingly.
